# The complete mitochondrial genome sequence of Lindian Chicken (*Gallus gallus*) in China

**DOI:** 10.1080/23802359.2018.1532336

**Published:** 2018-10-26

**Authors:** Yuan Xu, Enze Jiang, Chong Chen, Jiayu Liu, Keli Zhu, Zhen Zhang, Fangyong Ning, Zhiheng Du, Xiujuan Bai

**Affiliations:** College of Animal Science and Technology, Northeast Agricultural University, Harbin, China

**Keywords:** Lindian Chicken, mitogenome, conservation genetics

## Abstract

In this study, the first complete mitochondrial genome of Lindian Chicken (*Gallus gallus*) was sequenced in order to develop the mitogenome data for genus gallus. The complete mitogenome sequence is 16,785 bp in length, containing 37 genes (13 protein-coding genes, 2 ribosomal RNA, 22 transfer RNA genes, and one control region). The new sequenced complete mitogenome of Lindian Chicken will provide useful information for application in conservation genetics and evolution for this Near Threatened Chicken genomes.

Lindian Chicken (*Gallus gallus*), a native breed from Lindian County, Heilongjiang province, the Northeast of China, where the minimum temperature could dip to as low as −40 °C, is famous for its strong cold resistance and crude feed resistance traits (Chen et al. 2004; Wang et al. 2016). However, after 1980s, the number of Pure-Breed Lindian Chicken decreased sharply year by year because of the introduction of foreign breeds with a high growth rate and irrational use of Lindian Chicken germplasm resources, and breed degraded simultaneously, which made this excellent breed to become extinct. Mitochondrial DNA was a useful marker to trace back the origin of livestock species and had been widely used to reconstruct domestication patterns (Groeneveld et al. 2010; Lorenzo et al. 2015). To research the genetic resource of Lindian chicken, we reported the mitochondrial genome of Lindian Chicken for the first time.

The female adult sample of Lindian Chicken used for this study was collected from the Acheng campus of Northeast Agricultural University, Harbin, Heilongjiang Province, China, in 2018. The mitogenomes were amplified using 14 primers referenced from the Wuhua three-yellow chicken (*Gallus gallus*) (KM096864.1), and PCR products were determined by the method of Sanger sequencing. The mitogenome of Lindian Chicken is 16,785 bp in length and was deposited in the GenBank under accession number MH732978. The overall nucleotide composition was 30.3% A, 23.7% T, 32.5% C, and 13.5% G, with a total A + T content of 54.0%. It has 37 mitochondrial genes, including 2 rRNA (12S rRNA and 16S rRNA) genes, 13 protein-coding genes (PCGs), 22 tRNA genes, and a noncoding regions.

To validate the phylogenetic position of Lindian Chicken, a maximum-likelihood phylogenetic tree of 26 chicken breeds was constructed based on the complete mitochondrial DNA sequence. As shown in the phylogenetic tree (Figure 1), the 22 breed, including Lindian chicken, formed a monophyletic clade, Wuding Chicken, Cenxi classical three-buff chicken, Lverwu Chicken and Nandan Chicken formed into another clade. This can be explained by the chickens that are independently domesticated in various regions of the world (Eltanany and Distl 2010; Lorenzo et al. 2015; Yuan et al. 2015).

**Figure 1.**
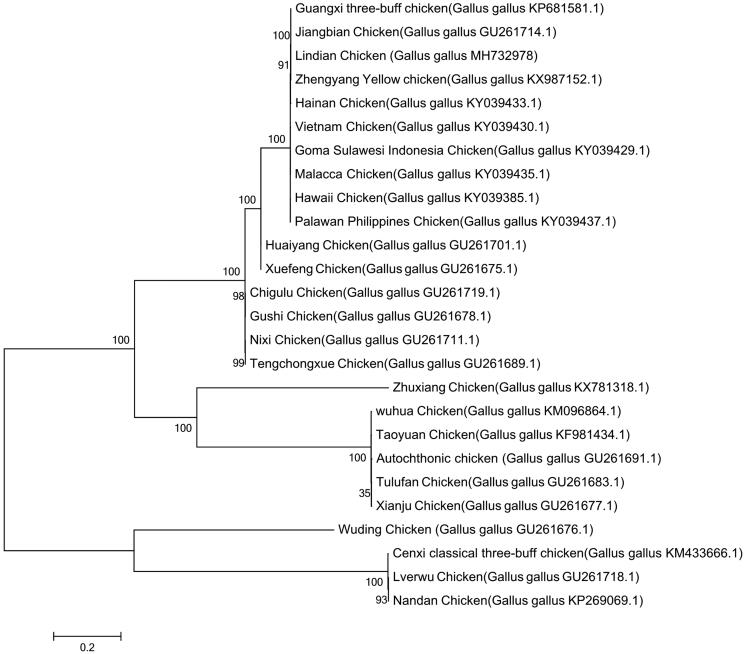
Maximum likelihood tree based on the complete mitochondrial DNA sequence of 26 chickens. Alphanumeric terms indicate the GenBank accession numbers.
